# Detecting Methylation Changes Induced by Prime Editing

**DOI:** 10.3390/genes16070825

**Published:** 2025-07-15

**Authors:** Ronin Joshua S. Cosiquien, Isaiah J. Whalen, Phillip Wong, Ryan J. Sorensen, Anala V. Shetty, Shun-Qing Liang, Clifford J. Steer

**Affiliations:** 1 Department of Medicine, University of Minnesota Twin Cities, Minneapolis, MN 55455, USA; cosiq001@umn.edu (R.J.S.C.); whale284@umn.edu (I.J.W.); wongx016@umn.edu (P.W.); lian0198@umn.edu (S.-Q.L.); 2 Department of Biochemistry, Molecular Biology, and Biophysics, University of Minnesota Twin Cities, Minneapolis, MN 55455, USA; soren940@umn.edu; 3 Department of Neurosurgery, University of Minnesota Twin Cities, Minneapolis, MN 55455, USA; shett098@umn.edu; 4 Department of Genetics, Cell Biology and Development, University of Minnesota Twin Cities, Minneapolis, MN 55455, USA

**Keywords:** CRISPR/Cas9, CpG, gene editing, methylation, off-target, prime editing

## Abstract

While prime editing offers improved precision compared to traditional CRISPR-Cas9 systems, concerns remain regarding potential off-target effects, including epigenetic changes such as DNA methylation. In this study, we investigated whether prime editing induces aberrant CpG methylation patterns. Whole-genome bisulfite sequencing revealed overall methylation similarity between Cas9-edited, and PE2-edited cells. However, localized epigenetic changes were observed, particularly in CpG islands and exon regions. The PE2-edited group showed a higher proportion of differentially methylated regions (DMRs) in some coding sequences compared to controls and Cas9-edited samples. Notably, CpG island methylation reached 0.18% in the PE2 vs. Cas9 comparison, indicating a higher susceptibility of these regulatory elements to epigenetic alterations by prime editing. Molecular function analyses including Gene Ontology and KEGG pathway analyses further revealed enrichment in molecular functions related to transcriptional regulation and redox activity in PE2-edited cells. These findings suggest that prime editing, while precise, may introduce subtle but functionally relevant methylation changes that could influence gene expression and cellular pathways. In summary, prime editing can induce localized DNA methylation changes in human cells, particularly within regulatory and coding regions. Understanding these epigenetic consequences is critical for the development of safer and more effective therapeutic applications of genome editing technologies.

## 1. Introduction

The development of prime editing over the past few years has yielded a form of genomic editing with increased precision [1] and avoids some of the issues that may arise from the double-stranded breaks inherent to the CRISPR-Cas9 genome editing [2]. The prime editing system involves the use of a pegRNA (prime-editing guide RNA), reverse transcriptase enzyme, and Cas9 nickase. The pegRNA guides the PE system to the target site on a gene of interest. The reverse transcription of an RT template on the pegRNA then results in the synthesis of a sequence of DNA with desired edits [1,3,4]. The resulting single-stranded flap of DNA then has the potential to hybridize with the original DNA strand, and through mismatch repair, the desired edited DNA sequence will be produced [1]. As a result, prime editing is a new and exciting development in the field of genetic editing that has the broad potential to be used in biotechnological and medical applications [1,4,5].

Despite the increased precision of genome modification technologies, some off-target effects can still be observed [6]. An example of an off-target effect is that of the increased methylation of CpG islands in regions that have undergone CRISPR-Cas9 modification [7]. CpG islands are DNA-rich regions that are rich in cytosine–guanine dinucleotides [8]. They typically reside in gene promoter regions, gene enhancer regions, or within genes themselves. The methylation of these CpG islands serves essential genomic functions in vivo, including regulating gene expression. However, off-target methylation due to editing within these regions can result in unintended gene expression alterations. These alterations can impact the efficiency of the edit and the effectiveness of the altered gene target [7]. Understanding the consequences of off-target effects in genomic editing technologies such as prime editing minimizes unintended genetic alterations, enhancing safety and precision in future therapeutic and research applications.

This study will seek to examine how genome editing through the use of prime editing systems results in increased CpG island methylation, similarly to that which was observed through the use of CRISPR-Cas9-mediated editing systems. CRISPR-Cas9 and prime editing systems will be used to induce a guanine (G)-to-thymine (T) transversion at *HEK4* site in HEK293T cells. Once the edit has been induced, methylation sequencing and various analysis methods will be completed using Reduced Representation Bisulfite Sequencing. Comparing the methylation patterns between the two genome editing technologies, CRISPR-Cas9 and prime editing, will provide the necessary information to determine whether similar off-target methylation is seen between the two.

## 2. Methods

### 2.1. Plasmids Used for CRISPR and Prime Editing

To generate pegRNA expression plasmids, gblocks or PCR products including spacer sequences, scaffold sequences, and 3′ extension sequences (pegRNA products from IDT) were amplified with indicated primers using Phusion master mix (Thermo Fisher Scientific, Waltham, MA, USA), which were subsequently cloned into a custom vector (Addgene plasmid no. 122089, BfuAI- and EcoRI-digested) by the Gibson assembly method (NEB). To generate sgRNA expression plasmids, annealed oligonucleotides were cloned into a BfuAI-digested vector [6]. All plasmids used for in vitro experiments were purified using a Plasmid Midi kit (Qiagen, Hilden, Germany), including an endotoxin removal step. pCMV-PE2 was a gift from David Liu (Addgene plasmid no. 132775) [1].

Spacers sequences of pegRNA: GGCACTGCGGCTGGAGGTGG

3′ extension sequences of pegRNA: AGAGTCTCCGCTTTAACCCCAACCTCCAGCC

Sequences of sgRNA: GGCACTGCGGCTGGAGGTGG

HDR template sequence:

5’TACTGCGTGGAGACAGACCACAAGCAGGTAAACAAGCAAATATGTAA-GTCCCAGGTCAGATAAATTTTAGGAAGTGCTGTTTTCCAGTGGTTCAATGGTC-ATCCCAGGGCAGAGGTGGGGAGACCTGCTGAGGGCGGCTTCTCCCTCAGTCA-GTCCATGCCTGCAGGGTCTGGAACCCAGGTAGCCAGAGACCCGCTGGTCTTC-TTTCCCCTCCCCTGCCCTCCCCTCCCTTCAAGATGGCTGACAAAGGCCGGGC-TGGGTGGAAGGAAGGGAGGAAGGGCGAGGCAGAGGGTCCAAAGCAGGATGA-CAGGCAGGGGCACCGCGGCGCCCCGGTGGCACTGCGGCTGGAGGGTTGTCCG-CTGTCACGACTCTGGGGGTTAAAGCGGAGACTCTGGTGCTGTGTGACTACAGTGGGGGCCCTGCCCTCTCTGAGCCCCCGCCTCCAGGCCTGTGTGTGTGTCTCCGT-TCGGGTTGAAAGGAGCCCGGGAAAAAGGCCCCAGAAGGAGTCTGGTTTTGGACGTCTGACCCCACCCCTCCCGCTTAGGGCTTCTGATCCCCCAGGGTGAT-3’

### 2.2. HEK293T Cells

HEK293T cells were grown in Dulbecco’s Modified Eagle Medium (DMEM) supplemented with 10% fetal bovine serum (FBS). The prime editing genome modification system (consisting of a pegRNA, spCas9, and plasmids) was transfected into the cells through the use of the Lipofectamine 3000 reagent system.

### 2.3. gDNA Isolation and Sequencing of Cas9-Edited and PE2-Edited Cells

gDNA was isolated from the transfected HEK293T cells through the use of the Qiagen DNeasy Blood and Tissue Kit [9]. gDNA from the Cas-9-edited, prime-edited, and control cells were PCR amplified using primers specific to the *HEK4* locus [1]. The amplified amplicons were then gel purified and sent for both Sanger Sequencing and NGS sequencing (2 × 250 bp paired end) to confirm editing efficiency.

### 2.4. Methylation Sequence

Isolated gDNA samples were analyzed via Reduced Representation Bisulfite Sequencing (RRBS). The data sequences were aligned and transferred into sequenced reads and analyzed in FASTQ file format. Methylation results were calculated by using the ASCII value of each character. If a test result yielded an error rate value, then the base mass of Illumina HiSeq X-ten was expressed as *Q_Phred_*.

$$Q_{Phred}=-10{log}_{10}(e)$$

Samples were then sequenced through the use of an Illumina HiSeq sequencing platform.

### 2.5. Methylation Analysis

The Bismarck software program (0.24.2) was used to statistically analyze the methylation sites. Methylation sites were detected through the alignment of the C-sites (Table 1, Table 2, Table 3 and Table 4).

Methylation level (ML)was calculated as follows:

ML=mC/(mC+umC)

Base methylation levels were calculated as follows:

Base ML=100×methylated reads/(methylated read+non methylated reads).

### 2.6. Calculation of Differentially Methylated Regions

For the identification of differentially methylated regions (DMRs), the R package methylKit (v. 3.22), specifically the getMethylDiff function, was used. The q value was set to <0.01 to control for false positives, and only regions with substantial biological relevance (methylation percent difference set to >25%) were used for the analysis.

### 2.7. Gene Ontology and KEGG Analysis of Differentially Methylated Regions

The list of genes based on DMR annotations were used for the Gene Ontology (GO) and KEGG analyses. Entries of observed DMR genes > 2, FoldChange > 2, and adjP < 0.05 were used for the GO analysis. The degree of KEGG enrichment was measured by the fold change, Qvalue, and the number of genes enriched into this pathway. Fold change referred to the ratio of the number of DMR-related genes enriched in the pathway to the number of annotated genes. The larger the fold change, the greater the degree of enrichment. Qvalue was the Pvalue after multiple hypothesis test correction. The Qvalue was [0,1]. The closer to zero, the more significant the enrichment. The 10 most significant path entries for enrichment were selected for the KEGG analysis figures.

## 3. Results

### 3.1. Cas9- and PE2-Based Editing of HEK293T Cells

**Figure 1:**
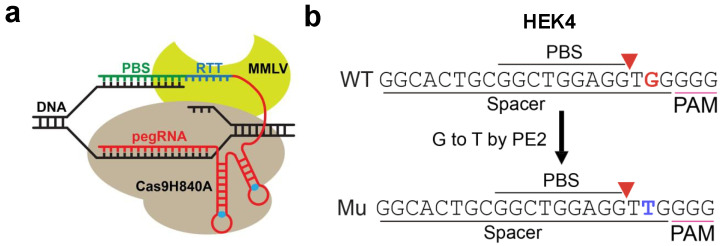
PE2 construct and overview of target site *HEK4.* (**a**) A prime editing complex consists of a PE protein containing an RNA-guided DNA-nicking domain, such as Cas9H840A nickase, fused to an RTT domain and complexed with a pegRNA. The PE-pegRNA complex facilitates a variety of precise DNA edits at a wide range of positions. (**b**) A G-to-T mutation is introduced by PE at *HEK4* locus.

HEK293T cells were transfected and edited using CRISPR-Cas9-based or PE2-based editing as described in the Methods section (Figure 1). To confirm the editing efficiency, amplified gDNA from the Cas-9-edited and PE2-edited conditions were sequenced using Sanger and NGS methods (Figure S4). For the Cas9 condition, the insertion at the *HEK4* locus was observed in both the Sanger and NGS sequencing with a high efficiency of 97.16%. The PE2-edited samples also displayed the intended ‘G’-to-‘T’ editing in the *HEK4* gene with an efficiency of 5.47%.

### 3.2. Whole-Genome Bisulfite Sequencing for a Genome-Wide Evaluation of CpG Methylation Patterns

**Figure 2:**
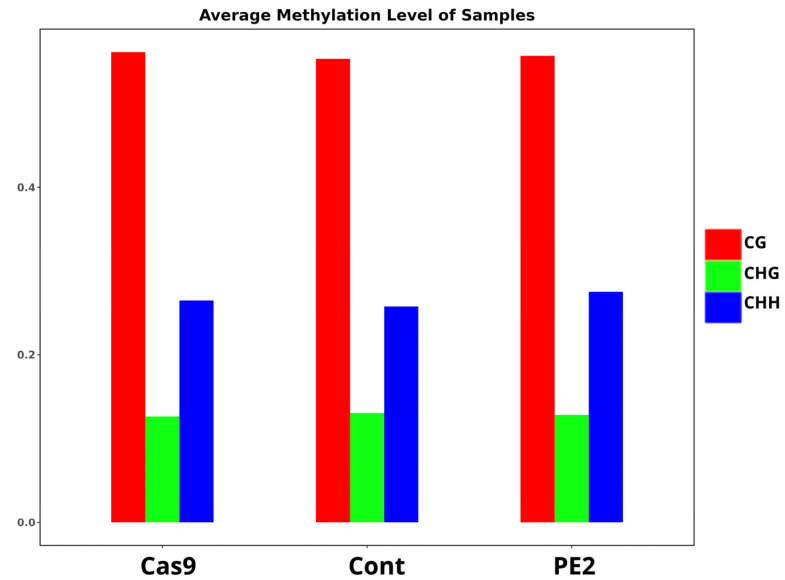
Average methylation level in whole genome. The methylation level of each methylated C base (CG, CHG, and CHH) was calculated. The C base is classified into three types according to the sequence features on the genome, namely CG, CHG, and CHH, where H represents A or T or C, respectively. This is calculated for each experimental trial (Cas9, control, and PE2) as follows: methylation rate of C site = 100* Support for methylated reads/(Support for methylated reads + Support for unmethylated reads). The genome-wide average methylation level reflects the overall characteristics of the genomic methylation profile.

The modifications induced within the methylome by the prime editor were evaluated using whole-genome bisulfite sequencing. The average methylation levels of the two edited groups (Cas9 and PE2) and their controls are described in Table 1. The prime-edited gDNA collected from the HEK293T cells were analyzed to assess CpG methylation following prime editing. To accomplish this, an Illumina HiSeq sequencing platform was utilized to generate high-throughput sequencing data from the cell samples that had undergone prime editing. This approach enabled a comprehensive examination of DNA methylation across the genome, facilitating the precise quantification of methylation levels at various cytosine sites. The study included three experimental groups: a control group, a Cas9-treated group, and a PE2-treated group. By comparing these groups, we aimed to determine whether prime editing or the use of Cas9 had any significant impact on DNA methylation patterns. Following sequencing, the average methylation levels of the three groups were computed and analyzed (Figure 2 and Figure S1). The focus was placed on three different types of cytosine methylation contexts: CG (CpG sites), CHG, and CHH, where H represents A, T, or C. The results demonstrated that the overall methylation levels across the control, Cas9, and PE2 groups were similar, suggesting that neither prime editing nor the presence of Cas9 led to drastic changes in DNA methylation. However, subtle differences were observed between the groups, indicating that while the methylation patterns remained largely conserved, minor variations existed. These findings suggest that while prime editing does not induce widespread methylation changes, it may still have localized effects on specific genomic regions, warranting further investigation.

**Figure 3:**
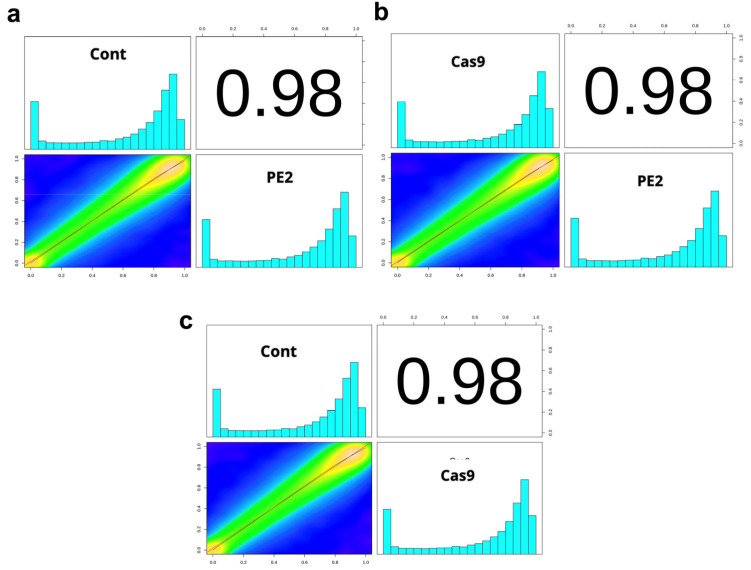
Correlation analysis of whole-genome methylation levels. The methylation level control vs. PE2 (**a**), PE2 vs. Cas9 (**b**), and control vs. Cas9 (**c**) in each bin was calculated using a 20 Kbp/bin subsequence environment, and then the Pearson Correlation Analysis was performed. Filtering was used to obtain methylation levels of CpG sites with a sequencing depth of 10× in all samples for this analysis.

Methylation correlations were then also calculated between the different experimental groups to assess the degree of similarity in DNA methylation patterns following prime editing (Figure 3). This analysis aimed to determine whether the introduction of the PE2 enzyme or the use of Cas9 led to significant deviations in methylation levels compared to the control group. Specifically, Figure 3a compares the control group with the PE2-treated group, Figure 3b examines the correlation between the PE2 and Cas9 groups, and Figure 3c compares the control group with the Cas9-treated group. The correlations of these three comparison groups were found to equal 0.98.

### 3.3. Differentially Methylated Regions

**Figure 4:**
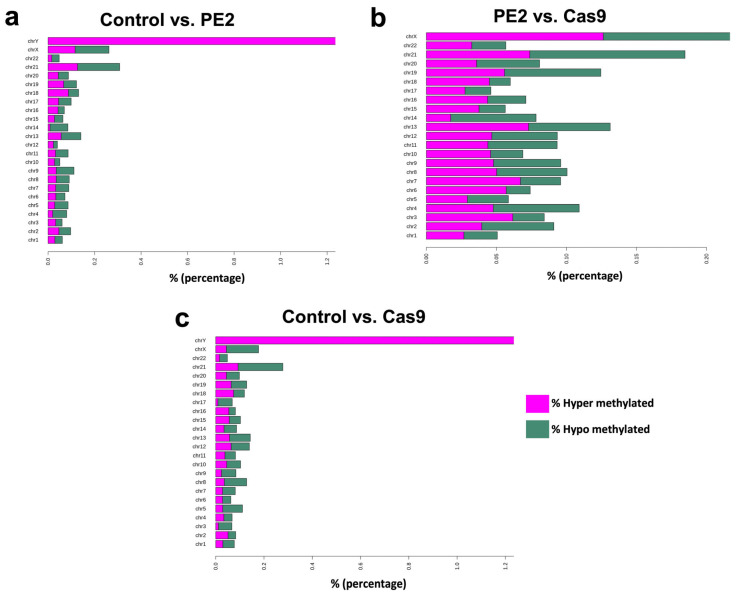
Differentially methylated region (DMR) distribution in each chromosome. In this analysis we used the methyl kit R package for DMR (differentially methylated region) analysis. Firstly, we take a sliding window scan method to calculate the methylation counts in a strict genome region for every sample, with a window size of 500 bp and a step size of 500 bp. Then the meth.diff parameter for each region is calculated for the control vs. PE2 (**a**), PE2 vs. Cas9 (**b**), and control vs. Cas9 (**c**). Finally, the DMR is obtained by multiple test calibration. The cutoffs for q-value and meth.diff parameters are 0.01 and 20%, respectively, which can be adjusted as required.

Differentially methylated regions (DMRs) were then analyzed and compared between the three experimental groups to assess whether prime editing or Cas9 treatment induced significant changes in DNA methylation at specific genomic loci. This analysis aimed to identify regions of the genome where methylation levels differed significantly between the groups, potentially revealing localized epigenetic alterations associated with the editing process. Figure 4a represents the comparison between the control group and the PE2-treated group, Figure 4b compares the PE2-treated group with the Cas9-treated group, and Figure 4c illustrates the differences between the control group and the Cas9-treated group. The results indicated that while the overall methylation patterns remained highly correlated across the groups, variations in the percentage of hypomethylated and hypermethylated regions were observed across different chromosomes. The extent of these methylation changes varied depending on the comparison, with certain chromosomes exhibiting a higher proportion of differentially methylated sites than others (Figure 4).

**Figure 5:**
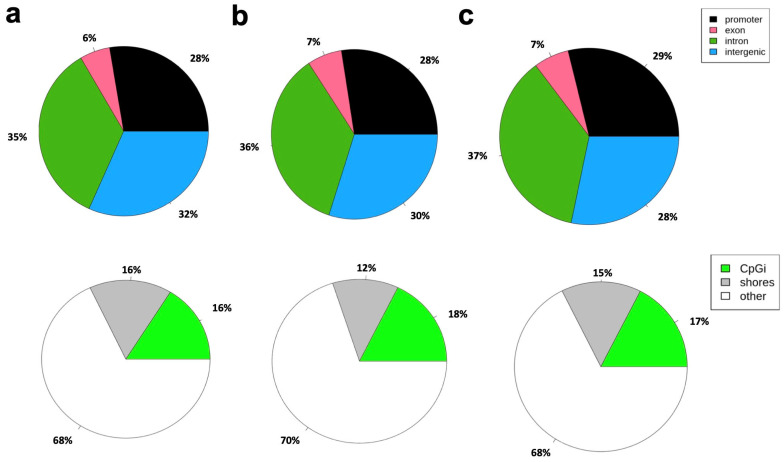
Genetic region annotation of differential methylated regions (DMR). (**a**) Control vs. PE2, (**b**) PE2 vs. Cas9, (**c**) Control vs. Cas9. The percentages of different genetic regions (up) and the percentages of CpG island regions (down) that relate to DMR in control vs. PE2 (**a**), PE2 vs. Cas9 (**b**), and control vs. Cas9 (**c**). Genetic region annotation for DMR (promoter, exon, intron, intergenic, etc.) based on transcript information of the reference species and CpG island region annotation to the DMR based on CpG island information of the reference species are applied.

Differential methylation was further examined across various genomic regions to determine whether prime editing or Cas9 treatment influenced methylation patterns in specific functional elements of the genome. This analysis involved comparing methylation differences in promoter regions, exons, introns, and intergenic regions among the three experimental groups: control, PE2, and Cas9 (Figure 5). Since DNA methylation in these regions can have distinct regulatory effects—such as influencing gene expression in promoters or affecting splicing in exons—this comparison provided insights into potential functional consequences of prime editing on the epigenome. Additionally, the methylation levels of CpG islands (CpGis), CpG shores, and other genomic locations were analyzed to assess whether prime editing affected methylation at these regulatory hotspots. CpG islands, which are frequently located near gene promoters, play a crucial role in transcriptional regulation, and changes in their methylation status can significantly impact gene activity. Shores, the regions flanking CpG islands, have also been implicated in gene regulation and epigenetic reprogramming. Notably, the comparison between PE2 and Cas9 (Figure 5b) revealed an increase in methylation levels specifically within exon regions, a trend that was not as pronounced in the control vs. PE2 or control vs. Cas9 comparisons. This finding suggests that prime editing using PE2 may lead to localized epigenetic modifications in coding regions, potentially affecting gene expression or alternative splicing. Furthermore, it was observed that among the three comparisons, the PE2 vs. Cas9 group exhibited the highest percentage of CpG island methylation, reaching 0.18% (Figure 5b).

### 3.4. Molecular Function

Molecular function enrichment analysis was conducted to compare the different treatment groups and identify potential functional pathways affected by prime editing or Cas9 treatment. This analysis aimed to determine whether specific biological processes or molecular functions were significantly enriched in response to genome editing. Two widely used bioinformatic tools were employed: Gene Ontology (GO) analysis and Kyoto Encyclopedia of Genes and Genomes (KEGG) pathway analysis. GO analysis focuses on categorizing genes based on their associated biological processes, molecular functions, and cellular components, while KEGG analysis provides insights into metabolic and signaling pathways that may be influenced by genetic modifications.

**Figure 6:**
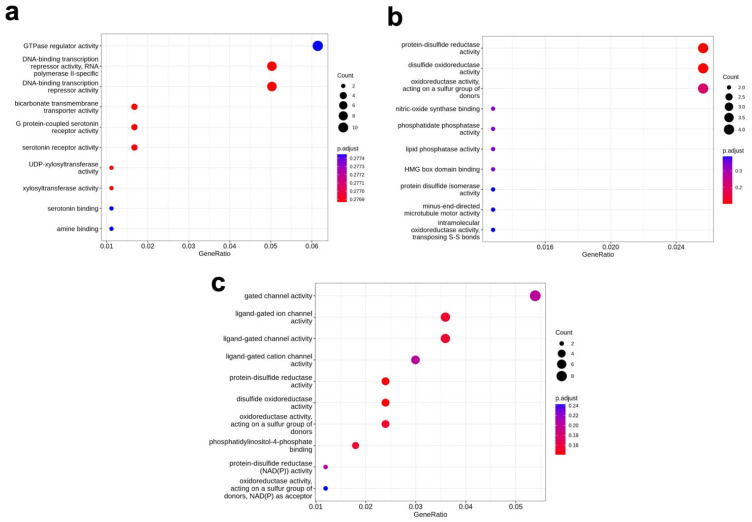
GO molecular function enrichment. The GO molecular function enrichment that relates to DMR in control vs. PE2 (**a**), PE2 vs. Cas9 (**b**), and control vs. Cas9 (**c**).

Using a Gene Ontology (GO) analysis, molecular function enrichment was assessed by calculating the gene ratio enrichment for different functional categories. In the control vs. PE2 comparison, the highest level of enrichment was observed in GTPase regulator activity, with a gene ratio greater than 0.06, suggesting that genes involved in this regulatory pathway were differentially affected by prime editing (Figure 6a and Figure S2). Following this, the next most enriched functional categories were DNA-binding transcription repressor activity and RNA polymerase II-specific DNA-binding transcription repressor activity, both of which exhibited gene ratios greater than 0.05. In control vs. PE2, the GO analysis of biological pathways displayed enrichment of terms related to glycolysis and NADH metabolic processes. Overall, when comparing PE2 vs. control, we observed enrichment for genes related to transcriptional regulation and redox activity.

In the PE2 vs. Cas9 comparison, enriched molecular functions were identified in protein-disulfide reductase activity and disulfide oxidoreductase activity, with gene ratios exceeding 0.024 (Figure 6b). For the control vs. Cas9 comparison, significant enrichment was observed in functions related to gated channel activity, with a gene ratio greater than 0.05. Specifically, ligand-gated ion channel activity and ligand-gated channel activity were enriched, both exceeding a gene ratio of 0.035 (Figure 6c).

**Figure 7:**
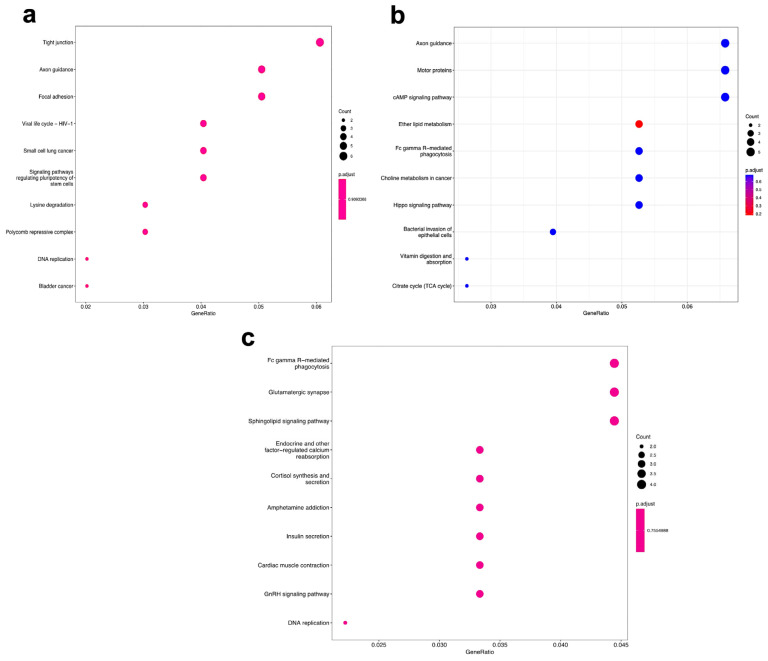
KEGG pathways of DMR-related transcripts in control vs. PE2 (**a**), PE2 vs. Cas9 (**b**), and control vs. Cas9 (**c**).

Using a Kyoto Encyclopedia of Genes and Genomes (KEGG) pathway analysis, gene enrichment was assessed across multiple biological processes, including signaling pathways, cellular processes, and genetic information processing (Figure 7 and Figure S3). The analysis revealed an increased number of genes associated with these pathways across all three experimental groups. We did not observe any common pathways/trends when comparing the pathways between PE2 vs. control, Cas9 vs. control, and PE2 vs. Cas9 (Figure 7 and Figure S4). However, a further analysis of the genes in the DMRs can provide more insight into regions targeted in different conditions.

## 4. Discussion

Prime editing represents a cutting-edge advancement in the field of genome editing, offering a novel approach that enhances the precision and specificity of genetic modifications [1]. However, unlike traditional CRISPR-Cas9 systems that rely on the introduction of double-stranded breaks, prime editing utilizes a more refined mechanism that facilitates target insertion, deletion, and base substitutions without the need for such breaks, thus reducing the risk of unwanted mutations [10,11,12]. This increased accuracy has positioned prime editing as a promising tool for a wide range of applications in biomedical technologies, therapeutic development, and medical research.

Despite its many advantages, prime editing is not without its drawbacks. One area of concern, as with other gene editing platforms, is the potential for off-target effects. These unintended alterations can manifest in various ways, including changes to DNA methylation patterns (which can influence gene expression and genomic stability). In this study, we identified potential epigenetic consequences of prime editing by examining methylation changes in CpG islands around prime-edited sites within HEK293T cells.

Our findings revealed notable differences in methylation patterns between cells that underwent prime editing methods compared to those modified using traditional CRISPR-Cas9 methods. Differences included both alterations to the quantity and distribution of methylated regions. Changes also included the identification of differentially methylated regions (DMRs) depending on the gene editing system that was employed (prime editing vs. CRISPR-Cas9). Further studies are required to determine the effect of those local epigenetic changes. Importantly, methylation variation was noted in exonic regions. Since exons play a critical role in protein coding, changes in their methylation status can have profound implications on gene expression and function. This further emphasizes methylation differences that occur when comparing prime editing to traditional CRISPR-Cas9 gene editing methods.

Overall, prime editing represents a powerful and promising tool in the realm of gene editing. However, despite its advantages, it is important that researchers be aware of the unintended epigenetic consequences (particularly off-site methylation effects) that may arise following its use. Similarly to observations made with the use of traditional CRISPR-CAS9 editing, prime editing results in the introduction of methylation changes (especially in CpG island regions). These changes have the potential to impact gene expression, which can have implications for the efficiency of prime editing systems and the use of prime editing in medical and biotechnological contexts. Ultimately, although prime editing holds great promise for the fields of biotechnology and medicine, more research is needed to further quantify the methylation changes incurred as a result of its use.

## Figures and Tables

**Table 1:** Average methylation level of each sample. For Cas9-edited (Cas9), Control (Cont) and PE2-edited (PE2) samples, the average methylation in different sites are listed.

Sample	CG_Sites	CG_Level	CHG_Sites	CHG_Level	CHH_Sites	CHH_Level
Cas9	8261436	0.561295	10172	0.126323	1102	0.264732
Cont	8028686	0.553411	10523	0.130532	1084	0.257863
PE2	8339041	0.556807	9706	0.128426	1083	0.275219

**Table 2:** Raw data statistic information. The number of reads, bases, and quality metrics for Cas9-edited (Cas9), Control (Cont) and PE2-edited (PE2) samples.

ID	Reads	Bases	Q20_Bases	Q20 (%)	Q30_Bases	Q30 (%)	GC_Content
Cas9	81731140	12259671000	11162724275	91.05	10046972287	81.95	42.35
Cont	72604456	10890668400	9980473084	91.64	9079741803	83.37	41.47
PE2	80107718	12016157700	10956798055	91.18	91.189860376079	82.06	42.85

**Table 3:** Methylation statistics in C site of whole genome. The methylation of the various cytosine sites in Cas9-edited (Cas9), Control (Cont) and PE2-edited (PE2) samples.

Sample	C	mC	mC %	CG	mCG	mCG %	CHG	mCHG	mCHG %	CHH	mCHH	mCHH %
Cas9	1438567163	122870303	8.54	252777071	119342131	47.21	387118516	1220866	0.32	798671576	2307306	0.29
Cont	1571793335	133611308	8.50	269031122	129803533	48.25	422583066	1309668	0.31	880179147	2498107	0.28
PE2	1428845943	121996982	8.54	249947085	118476765	47.40	384145186	1217365	0.32	794753672	2302852	0.29

**Table 4:** Statistics of DMR detection in the groups. The total DMRs, and percent of hyper- and hypo-DMRs in Cas9-edited (Cas9), Control (Cont) and PE2-edited (PE2) samples.

Group	Total_Regions	Total_DMRs	Hyper_DMRs	Hyper_DMRs (%)	Hypo_DMRs	Hypo_DMRs (%)
Cont_vs_Cas9	373527	538	0.14	220	0.06	318
Cont_vs_PE2	379227	493	0.13	221	0.06	272
Cas9_vs_PE2	371483	478	0.13	261	0.07	217

## Data Availability

Illumina sequencing datasets are available upon request. The authors declare that all data supporting the findings of this study are available within the paper and its Supplementary Information files. Backbone plasmids used for pegRNA and sgRNA cloning are available from Addgene. Source data are provided with this paper.

## Supplementary Materials

The following supporting information can be downloaded at: https://www.mdpi.com/article/10.3390/genes16070825/s1. Figure S1: Distribution of methylation site level. The methylation distribution in control (a), Cas9 (b) and PE2 (c). The horizontal axis (x-axis) is the methylation level and the vertical axis (y-axis) is the percentage of the C site in this sequence environment. Only the CpG context is considered. Figure S2: GO molecular function enrichment. The GO molecular function enrichment that relates to DMR in control vs. PE2 (a), PE2 vs. Cas9 (b) and control vs. Cas9 (c). Figure S3: KEGG pathway analysis. The KEGG pathways analysis that relate to DMR in control vs. PE2 (a), PE2 vs. Cas9 (b) and control vs. Cas9 (c). Figure S4: Sanger sequencing and NGS sequencing of Cas9-edited and PE2-edited samples, (a) Gel purification of amplified gDNA (control, Cas9-edited, PE2-edited) (b) Sanger sequenced gDNA results **c,** NGS amplicon sequenced gDNA results.
