# Supplementary material for: Detecting Methylation Changes Induced by Prime Editing
Source: Genes (Basel). 2025 Jul 15;16(7):825. doi: 10.3390/genes16070825 (PMC12294443; doi:10.3390/genes16070825)
Supplement: Supplementary file 1 [file genes-16-00825-s001.zip › Supplementary Figures.pdf]

Supplementary Information files. Backbone plasmids used for pegRNA and sgRNA cloning are available from Addgene. Source data are provided with this paper.

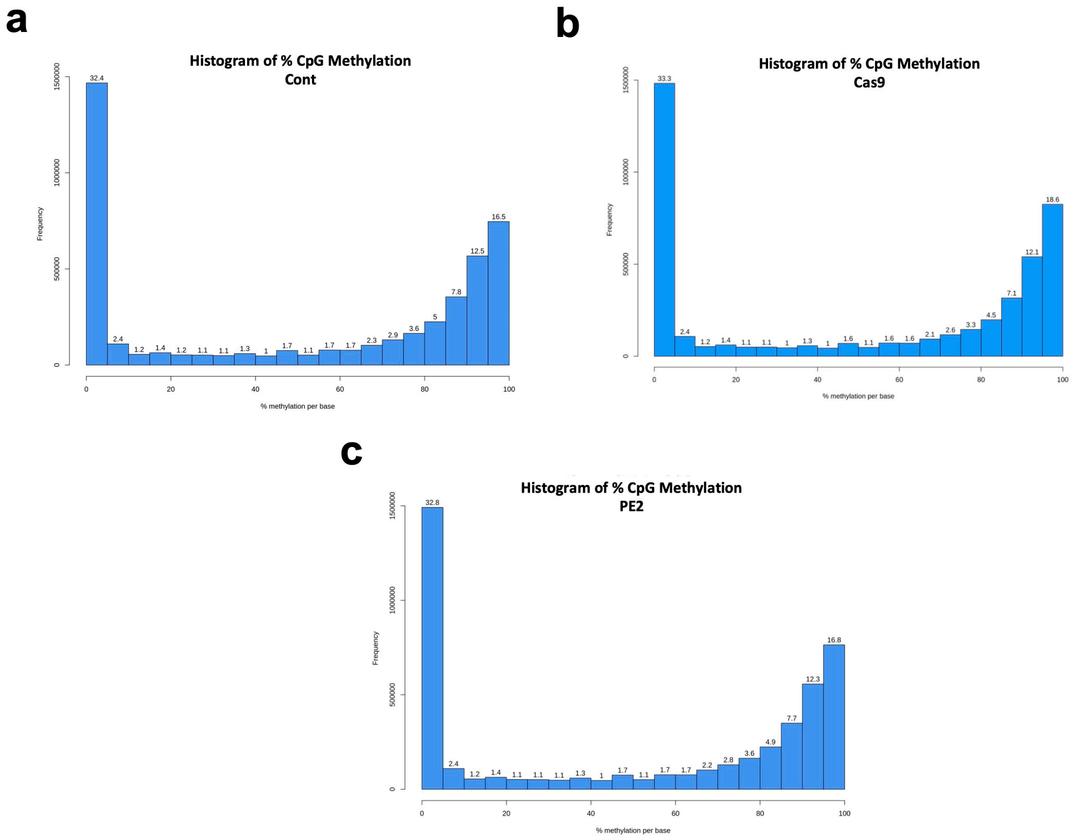

Supplementary Figure S1. Methylation distribution. The methylation distribution in control (**a**), Cas9 (**b**), and PE2 (**c**).

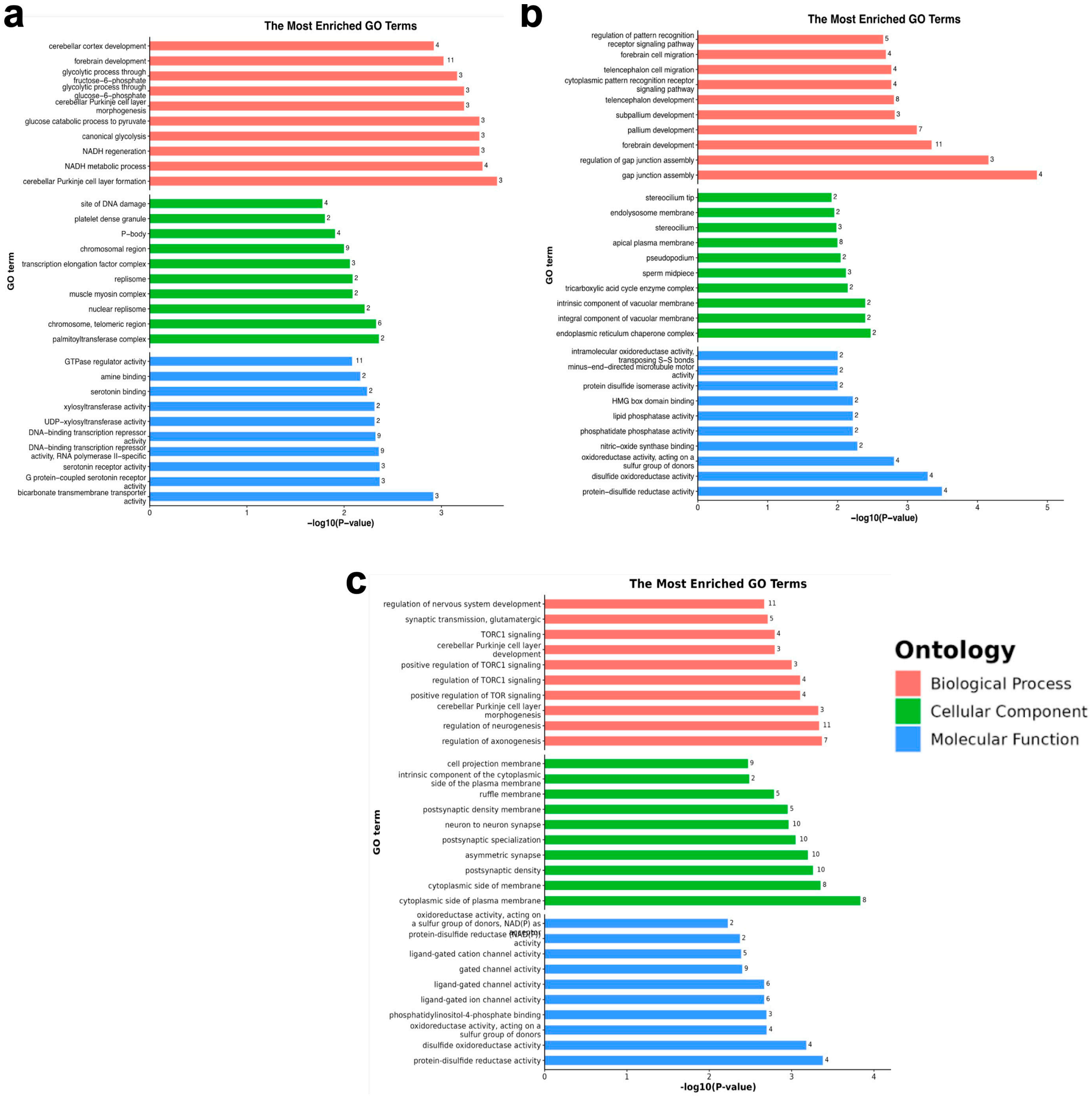

Supplementary Figure S2. GO enrichment of DMR in control vs. PE2 (**a**), PE2 vs. Cas9 (**b**), and control vs. Cas9 (**c**).

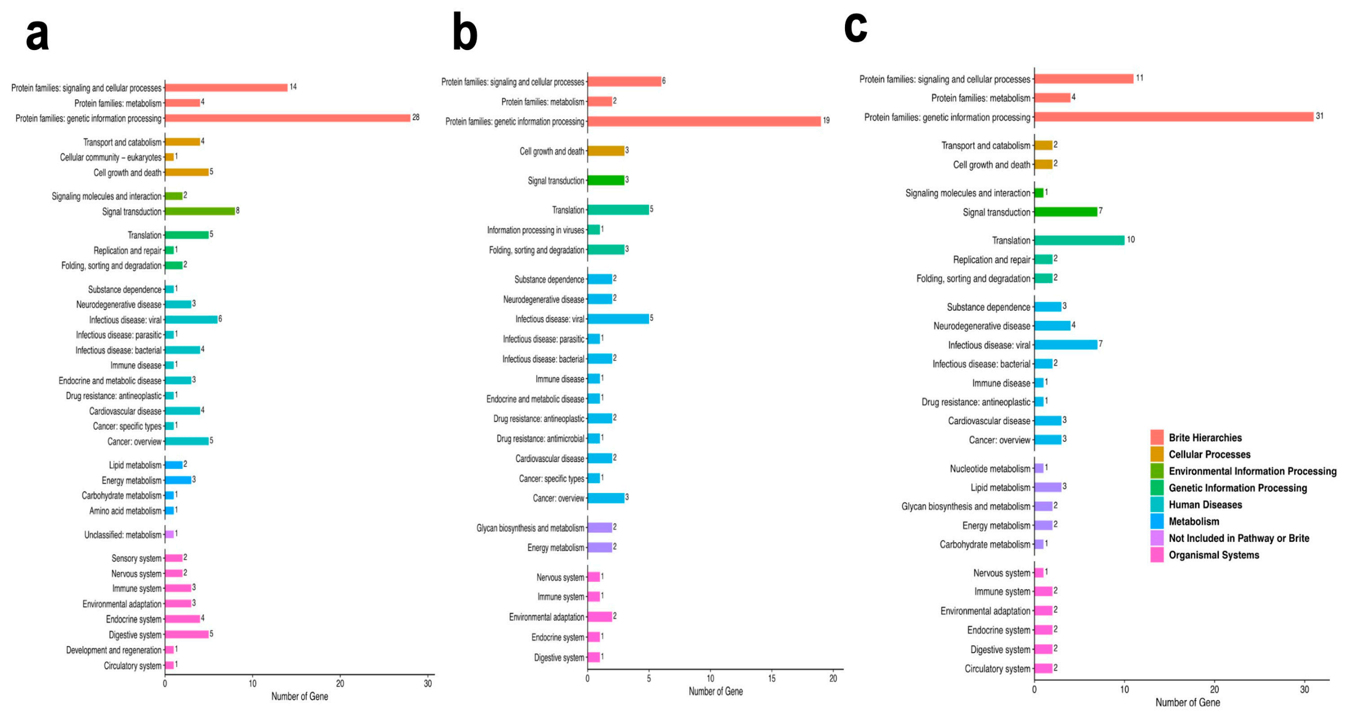

Supplementary Figure S3. KEGG enrichment. a) control vs. PE2; b) PE2 vs. Cas9; c) control vs. Cas9.

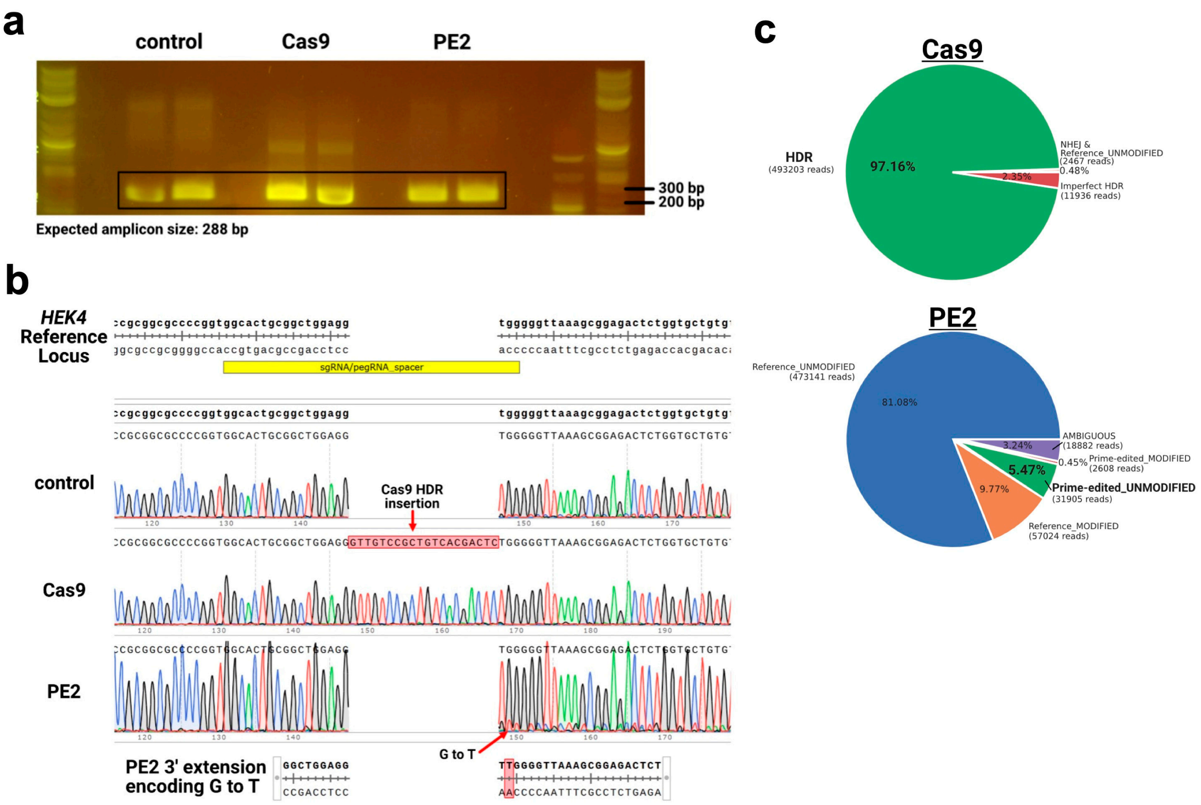

Supplementary Figure S4. Sanger sequencing and NGS sequencing of Cas9-edited and PE2-edited samples. (**a**) Gel purification of amplified gDNA (control, Cas9-edited, PE2-edited); (**b**) Sanger-sequenced gDNA; (**c**) NGS amplicon-sequenced gDNA.
