# Supplementary material for: Detecting Methylation Changes Induced by Prime Editing
Source: Genes (Basel). 2025 Jul 15;16(7):825. doi: 10.3390/genes16070825 (PMC12294443; doi:10.3390/genes16070825)
Supplement: Supplementary file 1 [file genes-16-00825-s001.zip › Supplementary Tables.pdf]

Table S1. Average methylation level of each sample

Sample	CG_sites	CG_level	CHG_sites	CHG_level	CHH_sites	CHH_level
Cas9	8261436	0.561295	10172	0.126323	1102	0.264732
Cont	8028686	0.553411	10523	0.130532	1084	0.257863
PE2	8339041	0.556807	9706	0.128426	1083	0.275219

Table S2. Raw data statistic information

ID	Reads	Bases	Q20_Bases	Q20(%)	Q30_Bases	Q30(%)	GC_content(%)
Cas9	81731140	12259671000	11162724275	91.05	10046972287	81.95	42.35
Cont	72604456	10890668400	9980473084	91.64	9079741803	83.37	41.47
PE2	80107718	12016157700	10956798055	91.18	9860376079	82.06	42.85

Table S3. Methylation statistics in C site of whole genome

Sample	C	mC	mC_percent	CG	mCG	mCG_percent	CHG	mCHG	mCHG_percent	CHH	mCHH	mCHH_percent
Cas9	1438567163	122870303	8.54	252777071	119342131	47.21	387118516	1220866	0.32	798671576	2307306	0.29
Cont	1571793335	133611308	8.50	269031122	129803533	48.25	422583066	1309668	0.31	880179147	2498107	0.28
PE2	1428845943	121996982	8.54	249947085	118476765	47.40	384145186	1217365	0.32	794753672	2302852	029

Table S4. Statistics of DMR detection in the groups

Group	Total_regions	Total_DMRs	Hyper_DMRs	Hyper_DMRs_Percentage(%)	Hypo_DMRs	Hypo_DMRs_Percentage(%)
Cont_vs_Cas9	373527	538	0.14	220	0.06	318
Cont_vs_PE2	379227	493	0.13	221	0.06	272
Cas9_vs_PE2	371483	478	0.13	261	0.07	217
